# Quiet as a Factor in the Treatment of Children

**Published:** 1884-07

**Authors:** Anna C. Howland

**Affiliations:** Poughkeepsie, N. Y.


### QUIET AS A FACTOR IN THE TREATMENT OF CHILDREN.

ANNA C. HOWLAND, M. D., POUGHKEEPSIE, N. Y.

[*Read before Homœopathic Medical Society of New York.*]

When an adult is seriously ill, particularly if he be nervous or sleepless, he is immediately put in a secluded room, with few going in or coming out, and if possible a nurse is employed; and all this is done often by the family themselves, without the advice of the physician. All see the importance of such measures. But the wee-bit babies are too often kept in the ordinary nursery (or wherever the cradle usually stands in the day-time), where very likely older children are running about, and all the cares and questions of every-day life are brought to the mother, as usual. Sometimes, in the families of the poor, it is impossible to insure quiet for old or young; but it is not for these I am now speaking. *Cœteris paribus.* A still room and systematic nursing will be obtained ten times for an adult where it is once for an infant. Because the baby cannot say that the noise or the light hurts, it is taken for granted that it does not; and many an incipient and otherwise curable brain-trouble is rendered fatal by this lack of care. I know many physicians attend to this important matter at once when called to see a sick child; but I also *know* that others do not. Too many are like one who was asked to write an article for this bureau, and refused from lack of interest in children. "In fact," he said, "he took no interest in them only as far as they brought in greenbacks." I wonder where this learned gentleman was made; he never *grew* surely; never had a childhood. But mothers should be taught the importance of immediately going into a comparatively dark room, where there are no flies, and no noise, when the baby is seemingly only a little ailing.

I remember distinctly being called a mile or two out of town one hot sultry August day to see a baby eighteen months old attacked with cholera infantum. It had been brought into town the previous day, and proper remedies administered by a homœopathic physician, who also gave the best advice as to the diet, but no directions regarding a quiet room. The child lay in a cradle, tossing and moaning, with eyes partly open; the vomiting was checked entirely, the bowels very much better, but the little worn-out sufferer was perishing for rest.

It was in the ordinary sitting-room; two older children were disputing over some game; another was practicing at intervals on the piano; somebody was sewing on a machine; two or three neighbors were giving advice, each, in her eagerness to be heard first, pitching her voice in its highest key; the hot air, laden with dust from the street, came pouring in at the open windows; flies innumerable were swarming in and the whole scene was one of dire confusion and discomfort. A warm bath, a darkened room, as far from the first as possible, due care taken, and proper remedies, changed the appearance marvelously in twenty-four hours, and the convalescence was sure though retarded by the previous uncomfortable surroundings.

Now these people were not poor, neither did they wish to kill the baby; but it always had slept in all this noise, and *nobody thought*—that was all.

And so I seriously believe many a little one is lost, just from this want of thought.

